# Allogeneic Lymphocytes Persist and Traffic in Feral MHC-Matched Mauritian Cynomolgus Macaques

**DOI:** 10.1371/journal.pone.0002384

**Published:** 2008-06-11

**Authors:** Justin M. Greene, Benjamin J. Burwitz, Alex J. Blasky, Teresa L. Mattila, Jung Joo Hong, Eva G. Rakasz, Roger W. Wiseman, Kim J. Hasenkrug, Pamela J. Skinner, Shelby L. O'Connor, David H. O'Connor

**Affiliations:** 1 Department of Pathology and Laboratory Medicine, University of Wisconsin—Madison, Madison, Wisconsin, United States of America; 2 Wisconsin National Primate Research Center, University of Wisconsin—Madison, Madison, Wisconsin, United States of America; 3 Department of Veterinary and Biomedical Sciences, University of Minnesota, Saint Paul, Minnesota, United States of America; 4 Laboratory of Persistent Viral Diseases, Rocky Mountain Laboratories, National Institute of Allergy and Infectious Diseases, National Institutes of Health, Hamilton, Montana, United States of America; New York University School of Medicine, United States of America

## Abstract

**Background:**

Thus far, live attenuated SIV has been the most successful method for vaccinating macaques against pathogenic SIV challenge; however, it is not clear what mechanisms are responsible for this protection. Adoptive transfer studies in mice have been integral to understanding live attenuated vaccine protection in models like Friend virus. Previous adoptive transfers in primates have failed as transferred cells are typically cleared within hours after transfer.

**Methodology/ Principal Findings:**

Here we describe adoptive transfer studies in Mauritian origin cynomolgus macaques (MCM), a non-human primate model with limited MHC diversity. Cells transferred between unrelated MHC-matched macaques persist for at least fourteen days but are rejected within 36 hours in MHC-mismatched macaques. Cells trafficked from the blood to peripheral lymphoid tissues within 12 hours of transfer.

**Conclusions/Significance:**

MHC-matched MCM provide the first viable primate model for adoptive transfer studies. Because macaques infected with SIV are the best model for HIV/AIDS pathogenesis, we can now directly study the correlates of protective immune responses to AIDS viruses. For example, plasma viral loads following pathogenic SIV challenge are reduced by several orders of magnitude in macaques previously immunized with attenuated SIV. Adoptive transfer of lymphocyte subpopulations from vaccinated donors into SIV-naïve animals may define the immune mechanisms responsible for protection and guide future vaccine development.

## Introduction

2.5 million individuals were infected with human immunodeficiency virus (HIV) in 2007. While antiretrovirals and improved education are critical to curbing the pandemic, the enormous number of new infections emphasizes the need for a prophylactic vaccine. However, two recent vaccine failures underscore that we do not yet have a firm understanding of the immunologic mechanisms responsible for control of viral replication [1]–[4]. These disappointing results intimate an urgent need for experiments that more accurately identify those immune responses that contribute to protective immunity and should be elicited by future vaccines.

Protective immune responses are evident in humans and macaques that spontaneously control HIV and simian immunodeficiency virus (SIV) replication, respectively. 0.3% of individuals have undectectable HIV plasma viremia in the absence of treatment [5], as do a comparable number of SIV-infected macaques [6]–[8]. Intensive genetic, immunologic, and virologic characterization of these individuals have failed to unambiguously identify the exact immune responses (or combinations of responses) that need to be elicited in a successful vaccine [9]. Cellular immune responses are clearly important, as evidenced by the 100–10,000-fold increase in plasma viral load following transient elimination of CD8+ T lymphocytes from SIV-controlling macaques [10].

These results provide hope that certain immune responses capable of controlling HIV and SIV exist, though eliciting these responses by prophylactic vaccination has proven enormously frustrating. Of all the vaccines tested to date, only attenuated ‘vaccine’ strains of SIV reliably and consistently elicit immune responses that control replication of pathogenic virus. However, this vaccine strategy is not safe for use in humans. Attenuated SIV is overtly pathogenic in neonatal macaques and sporadically pathogenic in adult macaques [11], [12], essentially confining the use of these vaccines to research studies of protective immunity. Therefore, the fundamental goal of ongoing studies of attenuated SIV is to define the immunological mechanisms responsible for protection, with the long-term aim of priming similar immune responses using safer vaccine modalities. However, extensive research to date has been unable to identify protective immune responses in macaques vaccinated with attenuated SIV.

The only retrovirus for which protective immune responses have been identified is friend virus (FV) [13]. FV is similar to SIV in that immunization with attenuated viruses protects from pathogenic virus challenge [14], [15]. Adoptive transfer experiments in mice delineated the need for a multifactorial vaccine-elicited immune response to protect against wildtype FV challenge. Neither CD8+ T cells nor CD4+ T cells alone prevented persistent infection after transfer to naive mice [16]. Instead, B cells, CD4+ T cells, and CD8+ T cells acted in concert to provide protection from persistent infection. Comparable adoptive transfer studies are needed to identify the minimal immune responses elicited by attenuated vaccines that control pathogenic SIV replication. Unfortunately, the genetic heterogeneity of the macaque major histocompatibility complex (MHC) has precluded using non-human primates in allogeneic adoptive transfer studies [17].

Previous adoptive transfer experiments in HIV and SIV studies have relied on autologous transfers or transfers between identical twins. These experiments have primarily focused on reconstituting the immune system in HIV+ persons or on testing the therapeutic effects of transferring SIV-specific cytotoxic T cells expanded *in vitro* [18]–[24]. Studies have also demonstrated the ability for peptide pulsed autologous cells to induce enhanced SIV and HIV specific T cell immunity in addition to recirculation of the labeled cells [25]. Additional studies have attempted selectively expanding different subsets of memory T cells, using different methods to expand cells, or transducing cells to express HIV-1 specific TCR chains [26]–[28]. These experiments using cultured cells have met with limited success due to the rapid clearance or death of transferred cells [19], [29]–[32]. However, none of these studies have been capable of investigating the mechanisms responsible for controlling viral loads in the context of live, attenuated vaccination. Several investigators have cited the lack of an adoptive transfer system in macaques to study protective immunity as a major obstacle to the development of an effective HIV vaccine [33], [34].

We recently identified a geographically isolated population of cynomolgus macaques from the Indian Ocean island of Mauritius (MCM) that have extremely limited MHC diversity [35]. We hypothesized that allogeneic lymphocytes would persist when transferred between feral, or wild caught, 2-MHC-haplotype-matched unrelated MCM, as these animals are MHC class I and II identical. Since only seven haplotypes account for all MHC diversity in MCM, pairs of MHC-identical animals can be easily defined using a panel of polymorphic microsatellite markers [35]. For example, an MCM that has haplotypes **H1/H2** is 2-MHC-haplotype-matched (completely MHC matched) to an **H1/H2** MCM, 1-MHC-haplotype-matched to an **H1**/H3 MCM and 0-MHC-haplotype-matched (mismatched) to an H3/H4 MCM. MCM offer the unprecedented opportunity to explore protective immunity elicited by attenuated vaccines through adoptive transfer of lymphocytes from vaccinated macaques into naive MHC-matched macaques followed by pathogenic challenge. Here we demonstrate that allogeneic lymphocytes persist for at least 14 days in 2-MHC-haploypte matched recipients and demonstrate that lymphocytes traffic to lymphoid tissues after transfer, confirming the unique suitability of MCM for adoptive transfer studies and establishing a framework for deciphering the correlates of SIV-specific protective immunity.

## Results

### Microsatellite genotyping identifies MHC-identical, MLR-nonresponsive MCM

**Figure 1:**
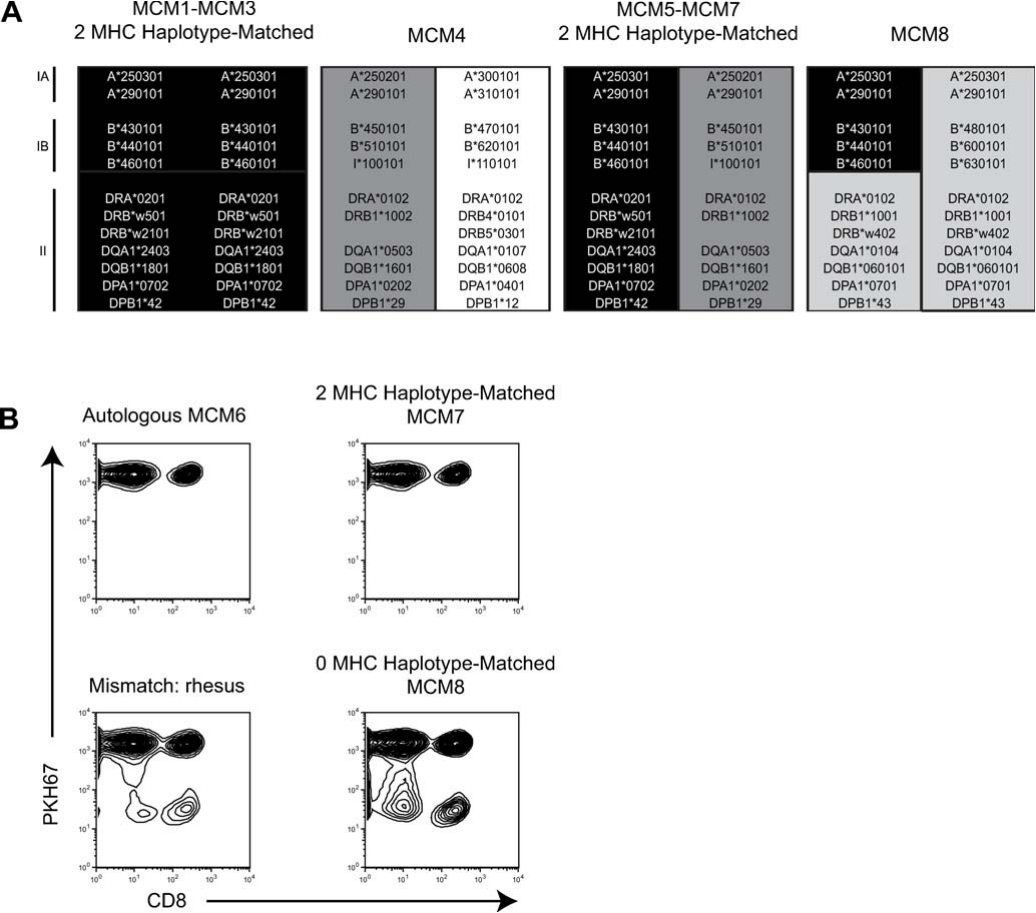
*In vitro* histocompatibility of MHC-matched MCM. A Microsatellite analysis was used to identify 2-MHC-haplotype-matched, 1-MHC-haplotype-matched, and 0-MHC-haplotype-matched MCM. MHC haplotypes inferred by microsatellite typing [35] are colored to show matching between individuals. Predicted MHC class I and class II alleles for each haplotype are shown. We identified 8 MCM for use in these experiments. B Results from mixed lymphocyte reactions. Whole PBMC from MCM6 was labelled with fluorescent dye PKH67 and incubated with irradiated stimulator cells as indicated above each plot. Cells that divide after recognizing non-self antigen lose fluorescence as indicated by a decrease along the y-axis. Dot plots are gated on lymphocytes according to forward and side scatter.

Eight MHC-defined MCM were used in this study. The animals were subdivided into two groups of three animals (MCM1-MCM3 and MCM5-MCM7) that are 2-MHC-haplotype-matched. MCM4 and MCM8 were included as 0-MHC-haplotype-matched controls (Fig. 1A). The predicted MHC class I and class II alleles encoded on each haplotype were defined in previous studies [35], [39].

Initially, the histocompatibility of 2-MHC-haplotype-matched MCM was assessed *in vitro* using a modified mixed lymphocyte reaction (MLR). Peripheral blood mononuclear cells (PBMC) from MCM6 were labelled with PKH67, a fluorescent cell membrane dye that segregates evenly during cell division [40], [41]. A reduction in PKH67 intensity indicates cellular proliferation. PKH67-labelled PBMC from MCM6 were cocultured with irradiated autologous, 2-MHC-haplotype-matched (MCM7), 0-MHC-haplotype-matched (MCM8), and 0-MHC-haplotype-matched rhesus macaque PBMC. PKH67 staining of CD8+ and CD4+ lymphocytes from MCM6 were analyzed after six days. Both CD8+ lymphocytes (Fig. 1B) and CD4+ lymphocytes (data not shown) from MCM6 proliferated when incubated with irradiated PBMC from either an Indian rhesus macaque or the 0-MHC-haplotype-matched MCM8. In contrast, the proliferation observed when labelled PBMC from MCM6 were incubated with irradiated PBMC from 2-MHC-haplotype-matched MCM7 was similar to the proliferation observed with irradiated autologous cells (Fig. 1B). We observed similar results in other pairs of 2-haplotype-matched MCM (data not shown). This encouraging *in vitro* data suggested lymphocytes would be tolerated when transferred between 2-MHC-haplotype-matched allogeneic MCM *in vivo*.

### Autologous CD8β+ lymphocyte transfer in MCM

We chose to focus our initial studies on the transfer of CD8β+ T cells. CD8β is frequently used as a marker for CD8 T cells that are both CD3+ and CD8+ [42]. CD8β+ T cells are archetypical of the refined lymphocyte subpopulations that could be examined in allogeneic transfer experiments. Previous experiments depleting CD8+ cells with monoclonal antibody have not distinguished between effects on the CD8+ T cell and CD8+ natural killer cell compartments, so understanding the role of CD8β+ T cells alone in SIV control could significantly advance our understanding of protective immunity [43]. Additionally, since CD8β+ T cells comprise approximately 10–20% of total lymphocytes in the blood, successful transfer of these cells would strongly suggest that other surface marker-defined lymphocyte subpopulations at comparable frequencies, such as CD4+ cells, B cells, and NK cells, or immunodominant CD8+ epitope-specific T cells, could be transferred alone or in combination from a donor animal and subsequently tracked in the recipient [44], [45].

**Figure 2:**
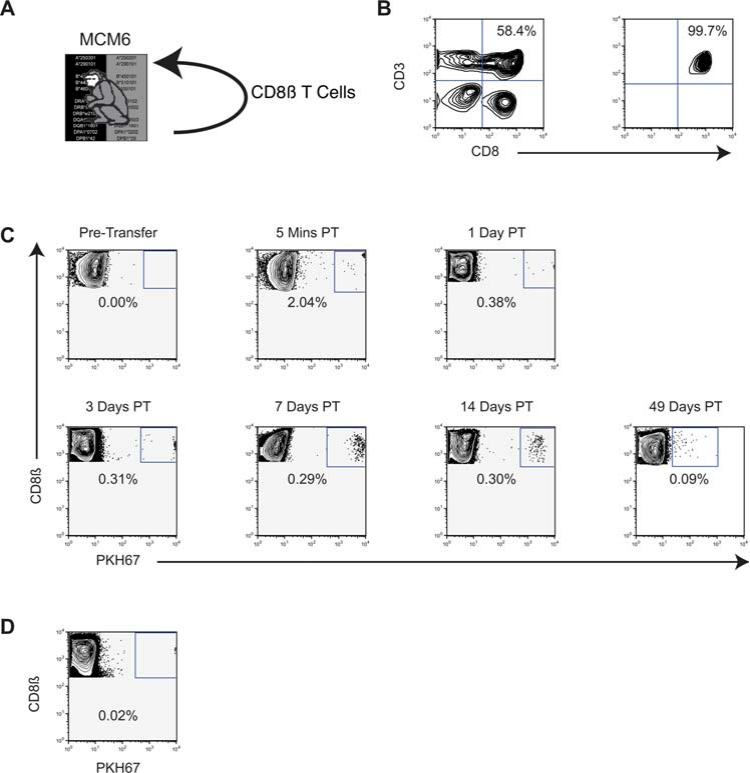
Autologous adoptive transfer. A Diagram displaying the transfer protocol. B Selection for CD8ß+ cells. The left panel is unmanipulated PBMC. The right panel is the positive fraction collected following CD8ß enrichment gated on lymphocytes. C Transfused cell persistence after transfer. Percentages are of total CD8ß+ cells in the PBMC of the animal based on the lymphocyte gate. D PKH67+ cells in lymph node of animal MCM6. The right inguinal lymph node was processed three days post-transfer to measure persistence of PKH67+ cells. The percentage of PKH67+ cells is based on total CD8ß+ cells in the lymph node lymphocyte gate.

We initially performed an autologous transfer to assess CD8β+ T cell purification, fluorescent labeling, *in vivo* persistence of labeled CD8β+ T cells, and trafficking of labeled donor cells in blood (Fig. 2A). We magnetically purified CD8β+ T cells from MCM6 PBMC by staining with anti-CD8β-PE antibodies. We enriched for our stained cells using anti-PE microbeads and magnetic columns. We passed the cells over the columns twice and obtained greater than 99% purity for CD8β+ cells in the lymphocyte gate (Fig. 2B). We elected to label the purified CD8β+ T cells with PKH67 due to this dye's high fluorescent intensity which allows for prolonged *in vivo* tracking, an advantage over other commercially available labels such as CFSE. We reinfused the labelled cells into MCM6 approximately 8 hours after the initial blood draw through the saphenous vein, and we monitored the persistence of PKH67+ cells in MCM6 by flow cytometry. Five minutes post-transfer we drew blood from the femoral vein and the transfused cells accounted for approximately 2.0% of CD8β+ T cells in the blood (Fig. 2B). After twenty-four hours, this number declined to 0.38%. The percentage of PKH67+ CD8β+ T cells remained constant for the next 14 days (Fig. 2C). PKH67+ CD8β+ T cells were detectable at low levels in PBMC for 49 days post-transfer, demonstrating the stability of the labelled lymphocytes *in vivo*. We also harvested an inguinal lymph node three days post-transfer and found that PKH67+ cells accounted for 0.02% of CD8β+ cells in the lymph node (Fig. 2D). The presence of cells in the lymph node provided evidence of trafficking despite the *in vitro* manipulations.

### Allogeneic CD8+ lymphocyte transfer between 2-MHC-haplotyped-matched unrelated MCM

**Figure 3:**
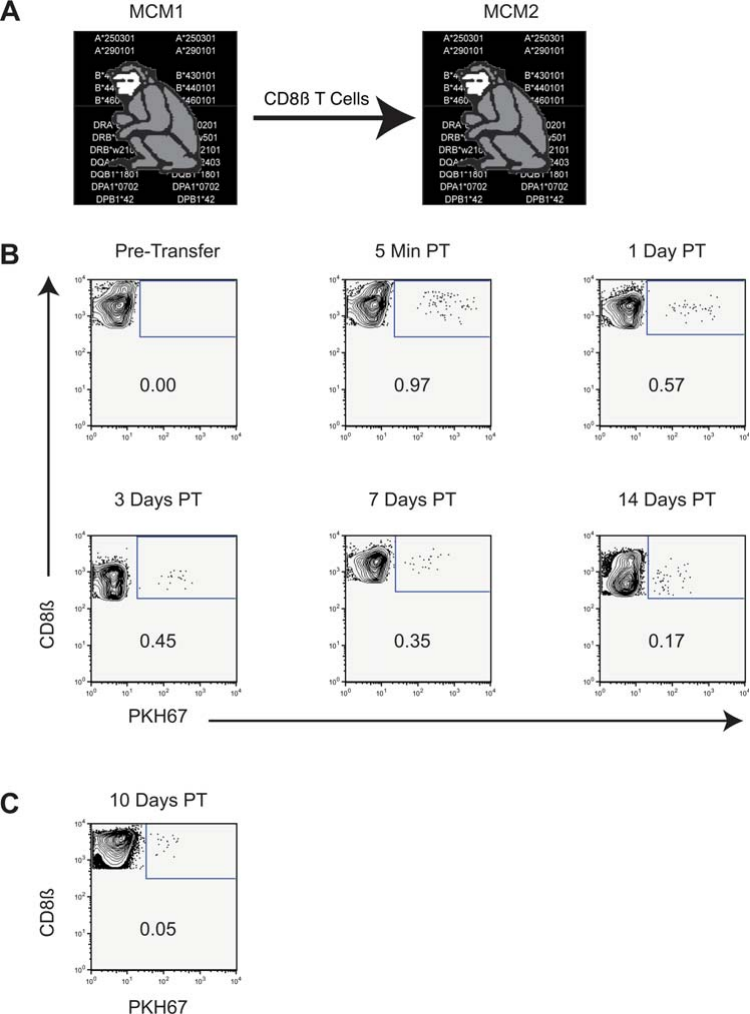
Allogeneic transfer from MCM1 to MCM2. A Diagram displaying the transfer protocol. B PKH67+ donor cells from MCM1 in PBMC of recipient 2-MHC-haplotype-matched MCM2. Dot plots are gated on lymphocytes and display CD8ß+ lymphocytes only. C Right Axillary lymph node sample 10 days post-transfer.

Building on the successful autologous transfer, we performed a CD8β+ T cell transfer between unrelated, 2-MHC-haplotype-matched MCM1 and MCM2 (Fig. 3A). We purified and PKH67 labelled CD8β+ T cells from MCM1 as described above and transferred them into MCM2. Five minutes after infusion into MCM2, the PKH67+ labelled cells accounted for nearly 1% of total CD8β+ T cells in the blood (Fig. 3B). Twenty-four hours post transfer, the PKH67+ labelled cells had decreased to nearly 0.6% of total CD8ß+ T cells in the blood. Transfused donor CD8β+ T cells persisted for at least 14 days post-transfer in MCM2. We also detected cells in the inguinal lymph node 10 days post transfer (Fig. 3C). To our knowledge, this is the first description of a successful allogeneic lymphocyte transfer between two unrelated, non-human primates without immunosuppressive therapy.

### 2-MHC-Haplotype Matched MCM Tolerate Adoptive Transfer of More than 2×10^8^ Lymphocytes

**Figure 4:**
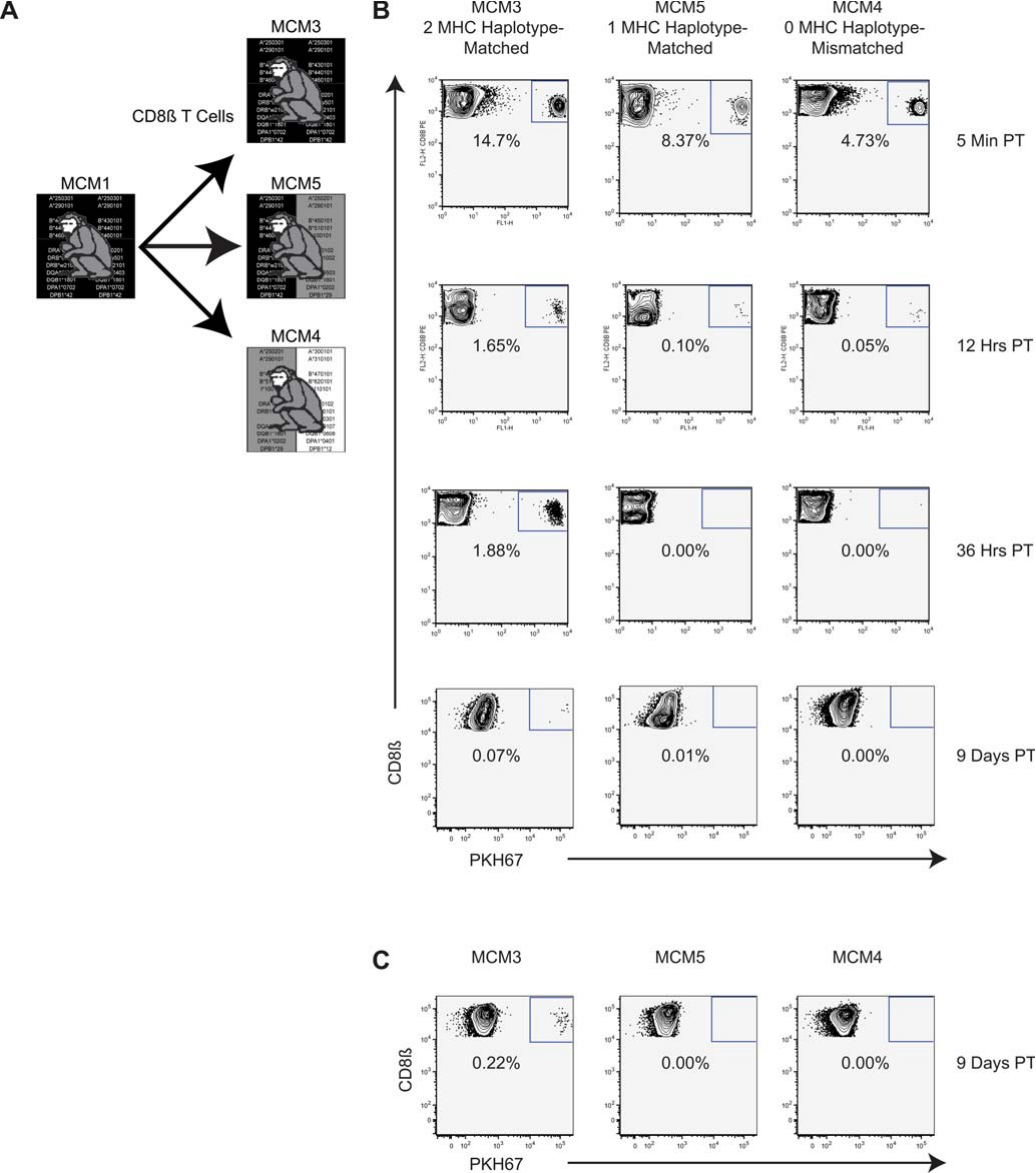
Allogeneic adoptive transfer from MCM1 to MCM3, MCM4, and MCM5. Gating strategy is described in the legend to Figure 2. A Diagram displaying the transfer protocol. B Donor CD8ß+ cells were transferred from MCM1 into 2-MHC-haplotype-matched MCM3, 0-MHC-haplotype-matched MCM4, and 1-MHC-haplotype-matched MCM5 and monitored for persistence in recipient PBMC. 220 million CD8ß+ were transferred to each animal. Donor cells were a composite of CD8ß+ cells enriched from PBMC, mesenteric lymph node and spleen. C Left inguinal lymph node sample 9 days post-transfer.

Next we sought to investigate how many lymphocytes could be safely transferred and whether persistence of transferred lymphocytes is MHC-dependent. We transferred CD8β+ T cells from an SIV+ donor into recipient animals that were 0-MHC-haplotype-matched, 1-MHC-haplotype-matched, and 2-MHC-haplotype-matched (Fig. 4A). We were particularly interested in determining whether an MHC homozygous animal would be an appropriate donor for a 1-MHC-haplotype-matched recipient as this could dramatically expand the number of recipient animals available for future transfer experiments.

Three weeks following intrarectal (IR) challenge with SIVmac239, we isolated over 6.5 billion cells from PBMC, spleen, and lymph nodes of MCM1 for transfer into animals MCM3, MCM4, and MCM5. We successfully isolated, labelled, and transferred 220 million CD8β+ cells into each of the three recipients. Transferred cells accounted for 4.7% to 14.7% of the CD8β+ cells in the PBMC of the recipient animals five minutes post-transfer (Fig. 4B) without any detectable adverse effects.

After 36 hours the donor cells were detectable only in the 2-MHC-haplotype-matched recipient, MCM3. The rapid elimination of PKH67+ CD8β+ T cells in the 0-MHC-haplotype-matched recipient (MCM4) and 1-MHC-haplotype-matched recipient (MCM5) underscores the unique suitability of 2-MHC-haplotype-matched MCM for allogeneic lymphocyte transfers. Hybrid resistance and missing-self killing by natural killer cells may explain the rejection of cells in the 1-MHC-haplotype-matched recipient [36]–[38]. The matched recipient had detectable viral RNA in plasma after the transfer suggesting the animal was infected by cell-associated virus (data not shown). This result demonstrates the importance of using vaccinated animals in future transfers.

The donor cells persisted for at least nine days in PBMC and lymph node of the 2-MHC-haplotype-matched animal MCM3 (Fig. 4C). PKH67+ cells accounted for a greater percentage of CD8β+ cells in the lymph node than they did in the blood. We believe the differences observed in the detection of cells in the PBMC between the first allogeneic transfer and this larger transfer can be explained by differences in the donor transfusate composition (Fig. 3 and Fig. 4). The PBMC only accounted for approximately 2% of the transfusate in the transfer to MCM3, MCM4, and MCM5; thus, it is likely that we transferred no more than 4 million CD8β+ cells from PBMC into each of the three recipient animals. Our data suggests that cells harvested from spleen and lymph node preferentially trafficked from the blood into peripheral lymphoid tissues after transfusion.

**Figure 5:**
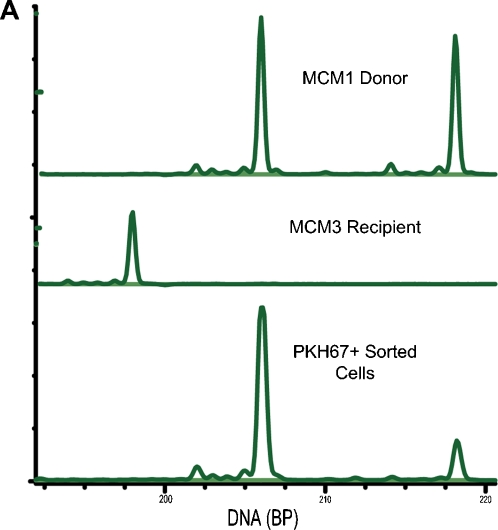
Allogeneic transfer cell analysis. A PKH67+ PBMC from recipient MCM3 were sorted from total PBMC by flow cytometry. We performed microsatellite analysis to demonstrate that the mircosatellite profile of PKH67+ cells in the recipient animal matched the microsatellite profile of donor MCM1.

In this transfer experiment we also verified that persisting PKH67+ CD8β+ T cells originated from donor MCM1. We took advantage of differences at microsatellite markers in non-MHC loci between donor MCM1 and 2-MHC-haplotype-matched, recipient MCM3. At 7 days post-transfer, we sorted PKH67+ cells from MCM3 PBMC, isolated genomic DNA, and performed microsatellite analysis. PKH67+ cells possessed the same microsatellite profile as the donor animal, MCM1 (Fig. 5A). These results confirm that the persisting PKH67+ CD8β+ T cells originated from the donor animal.

### Allogeneic bulk PBMC transfer between 2-MHC-haplotype matched unrelated MCM

**Figure 6:**
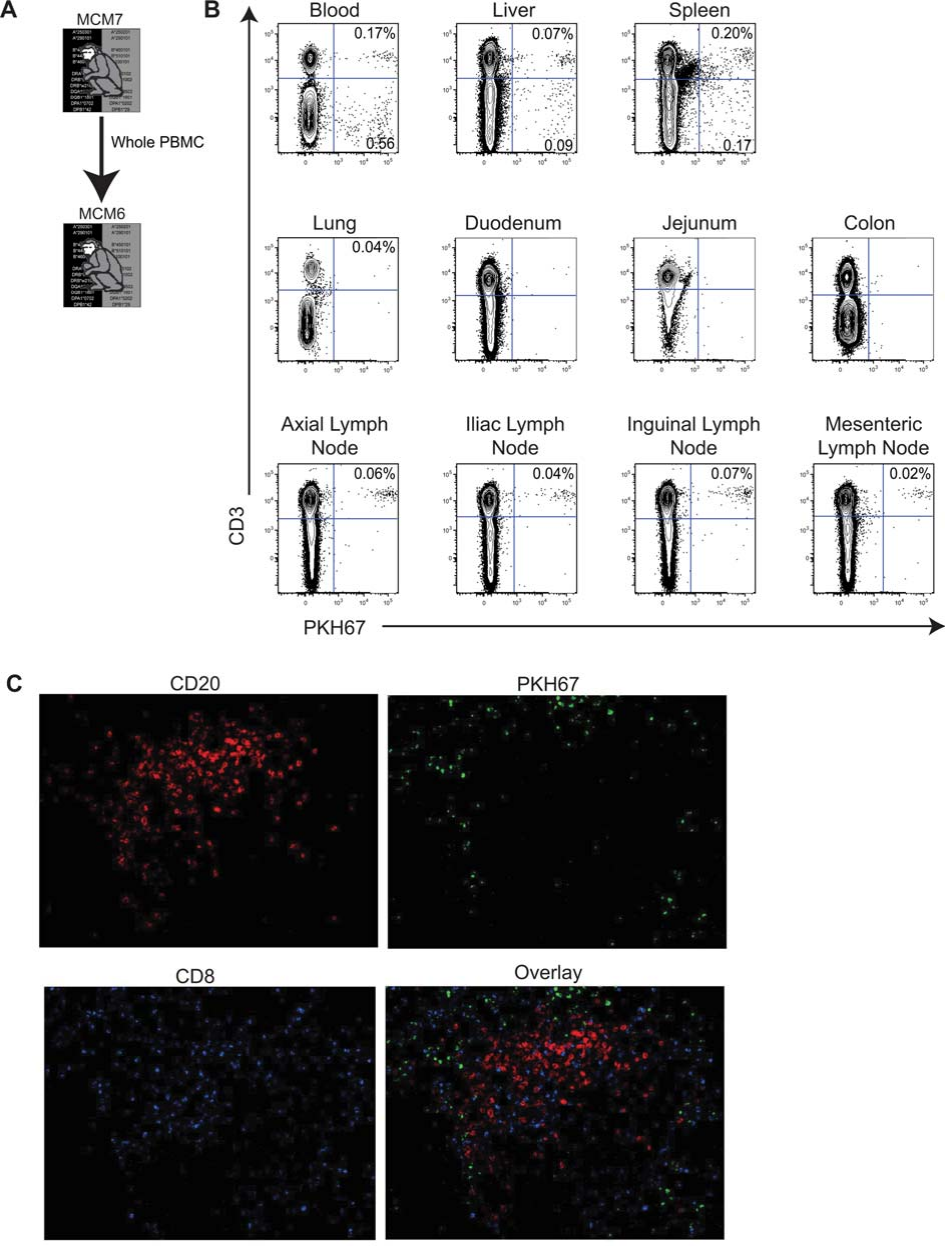
Lymphocytes traffic to peripheral lymphoid tissues after adoptive transfer. A Diagram displaying the transfer protocol. B Donor bulk PBMC were transferred from MCM7 into MCM6. MCM6 was euthanized 24 hours post transfer and its tissues were processed. Cells selectively trafficked to different peripheral lymphoid organs post-transfer. C Immunohistochemical staining of a lymph node section showing PKH67+ cells (green), CD20 antibody staining (red) and CD8 antibody staining (blue) collected with a confocal microscope at 200X.

We peformed another allogeneic transfer experiment between 2-haplotype-matched MCM to determine if cells were trafficking to additional tissues besides peripheral lymph nodes (Fig. 6A). Furthermore we used bulk PBMC in this experiment to monitor the trafficking pattern of different lymphocyte subsets. We transferred 60 million bulk PBMC from MCM7 into MCM6. Twenty-four hours post-transfer we euthanized MCM6 and examined tissue biopsies for the presence of PKH67+ cells. We found that CD3+ cells, but not CD3- cells, trafficked to lymph nodes (Fig. 6B). These CD3+ cells contained roughly equal proportions of CD4+ and CD8+ T cells (data not shown). Histology of the lymph nodes also showed the presence of PKH67+ cells (Fig. 6C). Both CD3+ and CD3- cells were detected in blood, spleen, and liver, including CD19+ B cells. PKH67+ cells were below the limit of detection in the mucosal tissues we sampled including, lung, duodenum, jejunum, and colon. While cells were not detected in the mucosal tissues, we only processed a small sample from each of these tissues. Donor cells were not harvested from the mucosal tissues which may have increased the presence of transferred cells in recipient mucosal tissues.

## Discussion

In these studies we describe a novel adoptive transfer model in macaques that can directly assess the contributions of cellular and humoral immune responses to protective immunity in macaques. In addition to being the first description of allogeneic adoptive transfers in nonhuman primates without immunosuppression, the successful transfer of bulk PBMC as well as specific lymphocyte subpopulations suggests that this system could be immediately applied to answer questions about the correlates of protective immune responses.

Differences at minor histocompatibility antigens may have limited the long-term *in vivo* persistence of transferred cells beyond two weeks. Nonetheless, our results clearly show that transfers between 2-MHC-haplotype-matched MCM are successful in a way that cannot be replicated in 1-MHC-haplotype-matched MCM or in other primate populations such as rhesus macaques where haplotype sharing between outbred animals is extremely rare [46].

In future experiments, it may be desirable to perform adoptive transfers without the need for fluorescent dye labeling. The development of non-fluorescent, genetic detection methods will help identify transferred cells when the dye is no longer detectable. Even though 2-MHC-haplotype-matched animals have identical genetics in the MHC loci, polymorphisms at other genetic loci can be used successfully to differentiate donor and recipient lymphocytes. For example, our laboratory has developed a panel of PCR-SSP assays for killer-immunoglobulin receptor (kir) genes that differentiate MCM that are MHC-identical. The use of genetic markers should eliminate problems with inconsistent labeling and completely circumvent cell viability or functionality issues introduced by dye labeling, even though *in vitro* cyotkine detection assays suggest that the PKH67 labeling does not obviously affect cellular function (data not shown).

Provocative research questions that have previously been the exclusive purview of inbred animal models that are distantly removed from humans can now be addressed in the macaque model. The histocompatibility of allogeneic lymphocytes also suggests that 2-MHC-haplotype-matched MCM could be used for preclinical testing of differentiated stem cell therapies. Additionally, with macaques being used extensively as models for a plethora of human health studies besides AIDS, including SARS, tuberculosis, biodefense, transplant and cancer research; 2-MHC-haplotype-matched MCM offer enormous opportunities to rapidly advance our understanding of protective immunity.

Yet perhaps the most immediate application of this model is in AIDS research. The lack of a nonhuman primate allogeneic lymphocyte transfer model has been repeatedly cited as a major obstacle to HIV vaccine development [33], [34]. Adoptive transfers present a straightforward approach to defining the correlates of protective immunity. Attenuated SIV vaccine strains have been effective at protecting against wild type SIV challenge. However, the precise mechanisms driving this protection remain unclear. Lymphoctyes from animals vaccinated with attenuated SIV could be transferred to naive recipients shortly before wild-type SIV challenge. In the last two years, two prerequisites for these types of studies have been fulfilled. First, We showed that MCM are susceptible to infection with the highly pathogenic SIVmac239 strain that is often used as a challenge following attenuated vaccination [35]. Second, MCM immunized with live, attenuated SIVmac239Δnef before exposure to SIVmac239 exhibit a 3–4 log_10_ blunting of peak acute SIV viremia (data not shown). This difference in acute phase peak viral load provides a measurable indicator of protection in future adoptive transfer experiments, essentially establishing a 3–4 log_10_ reduction in viral load as the benchmark for success, irrespective of whether the transferred cells are bulk lymphocytes, individual lymphocyte subsets, combinations of lymphocyte subsets, or *in vitro* manipulated lymphocytes. Since peak SIV viremia occurs approximately 14 days post-infection, coincident with the persistence of adoptively transferred lymphocytes, it is reasonable to speculate that adoptively transferred cells may influence peak viremia. Furthermore, if lymphocytes with protective attributes can be transferred between animals, it will be possible to compare the protection afforded by different vaccine modalities, accelerating the advancement of promising candidates through the vaccine pipeline. In these ways, the demonstration of successful allogeneic adoptive transfer in MCM may pave the way for the identification of the necessary immune responses that protect against SIV *in vivo*.

## Materials and Methods

### Animals and SIVmac239 Challenge

We purchased blood samples from feral MCM for genetic analysis (Charles River BRF, Houston, TS). Blood from 117 feral MCM was obtained for genetic analysis. Animal pairs were identified based on microsatellite analysis as described previously [35].

MCM1-MCM7 were challenged intrarectally with single doses of 50,000 TCID_50_ of molecularly cloned SIVmac239 Nef open virus [47]. Animal studies were approved by University of Wisconsin Institutional Animal Care and Use Committee (IACUC). SIV-infected animals were cared for according to the regulations and guidelines of the IACUC by members of the Wisconsin National Primate Research Center (WNPRC).

### Whole Genome Amplification and Microsatellite Analysis

At 7 days post-transfer PKH67+ cells from MCM3 were sorted on a MoFlow cell sorter (DakoCytomation, Fort Collins, CO). DNA from approximately 600 cells was subjected to whole genome amplification (Qiagen RepliG Kit). Microsatellite analysis was performed with the whole genome amplified product from PKH67+ cells as well as genomic DNA prepared from donor MCM1 and recipient MCM3 PBMC prior to transfer essentially as described previously [35]. The following PCR primers were used in this analysis: D3S3045-F, 5′-ACCAAATGAGACAGTGGCAT and D3S3045-R, 5′-ATGAGGACGGTTGACATCTG. D3S3045-F was fluorescently labeled with HEX (6-carboxy-2′, 4, 4′, 5′, 7, 7′-hexachlorofluorescein) to facilitate detection of PCR products with an ABI 3730xl DNA Analyzer (Applied Biosystems, Foster City, CA). The D3S3045 microsatellite marker was selected because it was found to be informative for the MCM1 donor cells and the MCM3 recipient animal.

### Mixed Lymphocyte Reaction

Blood was drawn into EDTA tubes. PBMC was isolated from EDTA treated blood by Ficoll-Paque PLUS (GE Health Sciences, Piscataway, NJ) density centrifugation. Stimulator cells were irradiated and resuspended at 2×10^6^ cells/ml. One ml of the stimulator cell suspension was added to each well of a 24 well plate. 2×10^6^ PKH67 (Sigma-Aldrich, Saint Louis, MO) responder cells were added to the wells in a 1∶1 ratio with the stimulator cells and plates were incubated for 6 days. Cells were harvested on day 6 and phenotyped and run on a BD FACSCalibur (Becton Dickinson Biosciences, San Diego, CA).

### Cell Transfers

Blood was drawn from the donor animal and PBMC was isolated from EDTA treated blood by Ficoll-Paque PLUS (GE Health Sciences, Piscataway, NJ) density centrifugation. Spleen and mesenteric lymph node were processed by dicing and forcing through a 100 uM filter (BD Biosciences). We proceeded to process cells as we did for the PBMC. Cells were then isolated by staining with an anti-CD8ß+ PE antibody, clone 2ST8.5H7 (Beckman Coulter, Fullerton, CA) then subsequently labelled with anti-PE microbeads, and subjected to magnetic separation with LS Columns (Miltenyi Biotec, Auburn, CA). Purified cells were labelled with PKH67 (Sigma-Aldrich) according to the manufacturer's protocol. Cells were resuspended in 3 to 5 mls of PBS containing 15u/ml sterile heparin (USB Corporation, Cleveland, OH). Cells were transfused into the saphenous vein by the animal care staff at the WNPRC. For the autologous transfer and first allogeneic transfer, we transferred 17 million cells and 10 million cells, respectively, using only PBMC isolated from the blood. In the second allogeneic transfer we transfused 60 million bulk PBMC. For the final allogenic transfer, cells from spleen, lymph node, and PBMC were combined into a cell suspension. This combination of cells was purified and labeled, and 220 million cells were transferred into each animal.

### Staining

Cells were phenotyped using the following antibodies and run on the BD FACSCalibur (BD Biosciences): CD3-APC clone SP34-2 (BD Biosciences), CD8ß-PE clone 2ST8.5H7 (Beckman Coulter); CD3-APC (BD Biosciences), CD8α-PerCP clone SK1 (BD Biosciences); CD3-APC (BD Biosciences), CD4-PerCP clone SK3 (BD Biosciences).

### Fresh tissue immunohistochemistry

Fresh lymph nodes were placed into cold RPMI containing 100ug/ml heparin and shipped on ice blocks overnight to Dr. Pamela Skinner's laboratory for fresh tissue sectioning and immunohistochemistry. Immediately upon arrival, the tissues were cut into 200 micron thick sections using a vibratome and placed in 24-well tissue culture plates with cold phosphate buffered saline containing 100mg/ml heparin (PBS-H). Free floating sections were incubated overnight on a rocking platform at 4°C with purified mouse-anti-human CD8 antibodies (Dako) diluted 1∶200 in a blocking solution of PBS-H containing 2% normal goat serum. The following day, sections were washed in PBS-H, fixed with 4% paraformaldehyde for 2 hours at room temperature, rinsed briefly, boiled in 0.1M Urea, and permeabolized and blocked in blocking solution containing 0.3% Triton X-100. The sections were then incubated at 4°C overnight with purified rabbit-anti-human CD20 antibodies (Lab Vision) diluted 1∶100 in blocking solution containing 0.3% Triton X-100. The sections were washed with PBS-H, and incubated at 4°C overnight with Cy3-conjugated goat anti-rabbit (1∶5000) and Cy5-conjugated goat anti-mouse antibodies (1∶2000) (Jackson ImmunoResearch) in blocking solution containing 0.3% Triton X-100. Finally, sections were washed in PBS-H and post-fixed with 4% paraformaldehyde, rinsed and mounted with glycerol gelatin (Sigma) containing 4 mg/ml n-proply gallate and examined and imaged using an Olympus FV 500 or 1000 confocal microscope.
